# Trust the Patient Not the Doctor: The Determinants of Quality of Life in Cervical Dystonia

**DOI:** 10.3389/fneur.2020.00991

**Published:** 2020-09-04

**Authors:** Ihedinachi Ndukwe, Sean O'Riordan, Cathal B. Walsh, Michael Hutchinson

**Affiliations:** ^1^Department of Neurology, St Vincent's University Hospital, Dublin, Ireland; ^2^School of Medicine and Medical Sciences, University College Dublin, Dublin, Ireland; ^3^Department of Mathematics and Statistics, University of Limerick, Limerick, Ireland

**Keywords:** cervical dystonia, non-motor symptoms, anxiety, depression, mood disorder, health-related quality of life

## Abstract

**Background:** Mood disorder is common in cervical dystonia and can impact on quality of life. It often precedes the onset of cervical dystonia and does not improve with botulinum toxin therapy.

**Objective:** To assess health-related quality of life in relation to mood disorder and measures of severity, disability and pain, in cervical dystonia patients receiving botulinum toxin therapy.

**Methods:** In a single-center, University Hospital movement disorders clinic, we conducted a comprehensive, cross-sectional study of disease severity, non-motor symptoms, mood and health-related quality of life in patients with cervical dystonia receiving botulinum toxin therapy using TWSTRS-2 for pain, severity and disability; Beck Anxiety Inventory and Beck Depression Inventory. We assessed all variables in relation to health-related quality of life assessed by Cervical Dystonia Impact Profile-58 and the Euro-QoL Utility Index.

**Results:** In 201 patients (136 women), mean age 61.5 years, significant determinants of impaired health related quality of life were: being a woman, reporting a history of anxiety or depression, prevalent pain, disability, anxiety and/or depression but not physician-assessed disease severity.

**Conclusion:** Patient-reported measures of pain, disability and, most markedly, mood disorder, are significant factors affecting quality of life; these were totally unrelated to the neurologist-rated measure of disease severity. Mood disorders, the predominant predictor of quality of life, were not addressed in the botulinum toxin clinic.

## Introduction

Dystonia is a movement disorder characterized by sustained or intermittent muscle contractions causing abnormal, often repetitive, movements, postures, or both (1).

Adult-onset idiopathic focal dystonia (AOIFD) is the most common form of dystonia; the most common phenotype is cervical dystonia (2). Although considered primarily a motor disorder, non-motor symptoms, especially mood disorders, with resulting impaired health related quality of life (HrQoL), are commonly observed in cervical dystonia (3–18). Mood disorders may precede the onset of the motor symptoms of dystonia (8), and do not improve with botulinum toxin therapy (3, 5); they are considered a primary feature of AOIFD, not secondary.

In cervical dystonia, disease impact is commonly measured by the validated Toronto Western Spasmodic Torticollis Rating Scales (TWSTRS-2) scales of Disability, Severity and Pain (19). The TWSTRS-2 Severity scale is physician-rated; the Disability and Pain scales are patient-reported. The TWSTRS-2 Total is used commonly as summary measure and has been shown to be responsive to change in botulinum toxin trials.

The Cervical Dystonia Impact Profile-58 (CDIP-58) is a commonly-used, multi-dimensional, disease-specific, patient self-report of HrQoL (20, 21). The inter-relationships between physician-assessed motor severity and patient-reported measures of disease impact and HrQoL have been seldom assessed. We aimed, in our cervical dystonia clinic population, to examine the inter-relationships between motor and non-motor symptom severity, using these validated instruments in relation to measures of mood disorder and HrQoL measured by both the CDIP-58 and a generic instrument.

## Materials and Methods

### Study Participants

The study participants had been diagnosed with adult-onset isolated idiopathic cervical dystonia (CD) according to standard diagnostic criteria by two experienced neurologists; they were attending our single-center Movement Disorders clinic for botulinum toxin therapy. We excluded patients with: (a) other forms of dystonia at onset (generalized, segmental, other focal dystonias), (b) other neurological disorders and comorbidities (cognitive impairment) that would confound assessment or preclude completion of questionnaires. All patients were assessed just prior to their next scheduled botulinum injection, usually 3 months after the last injection. Patients were recruited consecutively at the clinic; given the relatively large group, patients were recruited from July 2018 to January 2020 (roughly over 18 months).

### Methods

We collected basic demographic information, including current age, age at onset of cervical dystonia, duration of cervical dystonia, the presence of a history, and time of onset, of a medically diagnosed anxiety or depression (given by any medical practitioner), social history, and other medical background history.

### Measures

Pain, severity and disability were assessed using the revised Toronto Western Spasmodic Torticollis Rating Scales (TWSTRS-2); TWSTRS-2 Severity assessed by three experienced, trained, raters (IN, SO'R, and MH); TWSTRS-2 Pain and Disability reported by the participants; TWSTRS-2 Total was calculated (19). For current anxiety and depression assessment, we used the Beck Anxiety Inventory (BAI) and the Beck Depression Inventory-II (BDI-II) (22–25). For HrQoL measures, we used the disease-specific measure, Cervical Dystonia Impact Profile-58 (CDIP-58) (20, 21) and a generic assessment tool, Euro-Qol-Utility Index (EQoL-UV) (26). For all assessment tools, except the Euro-Qol-UV (Utility Index), higher scores indicate worse symptoms/impact; with the Euro-Qol-UV, the closer the number is to 1, the better the quality of life.

### Statistical Analysis

#### Power Analysis

The total population of cervical dystonia in Ireland determined in a previous epidemiological study from the group, was estimated as 410 patients (27). A sample size calculator, using 95% confidence level and a 5% confidence interval, indicates we should have at least 196 patients in our sample size (28). Thus, we consider that our sample size (201 patients) in relation to population size is adequately powered.

#### Statistical Analytical Methods

Means with standard deviation, medians and interquartile range (IQR) were used to describe the distribution of our dataset. We used the Mann-Whitney (M-W) test to compare the differences between two independent groups with dependent variables, given the non-Gaussian distribution of our data as evidenced by the Anderson-Darling, D'Agostin and Pearson, Shapiro-Wilk, and Kilmogorov-Smirnov tests. Significance level was defined as *p* < 0.05; there was no correction for multiple significance testing. The inter-relationship between variables was assessed by simple linear and multiple variable regression using Prism 8 (GraphPad). Separate analyses were performed: (1): in the total cohort, (2): by sex and (3): by a history of anxiety and/or depression prior to the study (medically diagnosed mood disorder vs. no medically diagnosed mood disorder).

## Results

### Study Participant Demographics

Two hundred and thirteen patients fulfilled the study criteria; 12 (6%) patients did not complete all assessments and thus 201 were included in the analysis; there were 136 women (68%) and 65 men (32%). Their mean (±SD) current age was 61.5 (±12.7) years; mean age at onset of CD was 43.6 (±12.8) years and mean duration of CD was 17.9 (±11.9) years (Table 1).

**Table 1:** Characteristics of cervical dystonia cohort by sex and history of mood disorder.

**Groups (*N*)**	**All (201)**	**Women (136)**	**Men (65)**	**History of mood disorder (79)**	**No h/o mood disorder (122)**
**CURRENT AGE (Y)**					
Mean (SD)	61.5 (12.7)	62.9 (12.2)	58.5 (13.3)	59.7 (12.4)	62.6 (12.8)
Median (range)	62 (31–90)	63 (33–90)	60 (31–86)	62 (33–90)	63 (31–90)
		*(ns)*		*(ns)*	
**AGE AT CD ONSET (Y)**					
Mean (SD)	43.6 (12.84)	**45.5 (12.8)**	**39.7 (12.1)**	**41.2 (12.0)**	**45.1 (13.2)**
Median (range)	44 (20–79)	**45 (20–79)**	**39 (20–65)**	**41 (20–66)**	**45 (20–79)**
		***(p = 0.006)***		***(p = 0.039*****)**	
**DURATION OF CD (Y)**					
Mean (SD)	17.9 (11.97)	17.4 (12.1)	18.8 (11.7)	18.5 (13.1)	17.5 (11.3)
Median (range)	16 (0.16-60)	15 (0.16–60)	17 (2–49)	18 (0.16–60)	16 (1.5–59)
		*(ns)*		*(ns)*	
**TWSTRS-2 TOTAL**					
Mean (SD)	30.4 (14.4)	**32 (15)**	**27 (12)**	**33.6 (15)**	**28.7 (13.9)**
Median (IQR)	30 (19, 40)	**31 (20,43)**	**26 (21,34)**	**34 (21, 46)**	**29 (19, 37.3)**
		***(p = 0.043)***		***(p = 0.03)***	
**TWSTRS-2 PAIN**					
Mean (SD)	13.6 (9.7)	14 (10)	12 (8.7)	14.9 (9.8)	12.8 (9.5)
Median (IQR)	14 (5, 20)	15 (5.3, 22)	13 (5.0, 18)	15 (8, 22)	14 (4, 19)
		*(p = 0.111)*		*(p = 0.15)*	
**TWSTRS-2 SEVERITY**					
Mean (SD)	10.4 (4.6)	11 (4.5)	9.8 (5.0)	10.8 (4.8)	10.1 (4.5)
Median (IQR)	11 (7, 13)	11 (8, 13)	10 (6, 12)	10 (7, 14)	11 (7, 13)
		*(p = 0.16)*		*(p = 0.65)*	
**TWSTRS-2 DISABILITY**					
Mean (SD)	6.5 (4.9)	6.9 (4.9)	5.8 (5.1)	**7.7 (5.1)**	**5.8 (4.8)**
Median (IQR)	6 (2, 10)	6.0 (3, 9.5)	5.0 (2, 9.5)	**7 (4, 11)**	**5 (2, 9)**
		*(p = 0.114)*		***(p = 0.0067)***	
**BECK ANXIETY INVENTORY**					
Mean (SD)	11 (11)	12 (11)	9.6 (11)	**16.8 (13.1)**	**7.1 (7.1)**
Median (IQR)	7 (3, 16)	8 (4, 17)	6.0 (2, 14)	**13 (8, 26)**	**5 (2, 9.3)**
		*(p = 0.07)*		***(p< 0.0001)***	
**BECK DEPRESSION INVENTORY**					
Mean (SD)	11.6 (10.4)	12 (10)	10 (10)	**17 (11.6)**	**7.9 (7.6)**
Median (IQR)	9.0 (3, 18)	11 (4, 19)	7.0 (3, 16)	**16 (8, 24)**	**6 (2, 12)**
		*(p = 0.09)*		***(p< 0.0001)***	
**UTILITY INDEX**					
Mean	0.712	**0.6873**	**0.7628**	**0.620**	**0.771**
(SD)	0.259	**(0.2708)**	**(0.2265)**	**(0.287)**	**(0.221)**
Median	0.789	**0.755**	**0.820**	**0.681**	**0.827**
IQR	0.611, 0.902	**0.586, 0.871**	**0.661, 0.932**	**0.435, 0.852**	**0.676, 0.932**
		***(p = 0.037)***		***(p< 0.0001)***	
**CDIP-58**					
Mean (SD)	32.4 (21.9)	**35 (23)**	**26 (18)**	**38.5 (22.2)**	**28.6 (22.2)**
Median (IQR)	31 (14, 47)	**35 (17, 49)**	**23 (12, 38)**	**37 (21, 51)**	**24 (11.8, 44)**
		***(p = 0.0088)***		***(p = 0.002)***	

*Study population characteristics: Demographic characteristics, disease impact and Quality of Life in all (a): 201 cervical dystonia patients, divided (b): by sex (136 women and 65 men) and (c): by a reported history of mood disorder (79 with and 122 without). Measures of dystonia impact by TWSTRS-2 Total, Pain, Severity, Disability, with measures of anxiety (Beck Anxiety Inventory), depression (Beck Depression Inventory) and quality of life (by the Utility Index and CDIP-58). Significant group differences are in bold. Women reported significantly worse TWSTRS-2 Total scores and worse quality of life. Patients with a history of mood disorder, at any time, had ongoing more severe TWSTRS-2 Total and Disability, ongoing more severe depression, anxiety and worse quality of life (TWSTRS, Toronto Western Spasmodic Torticollis Rating Scale; CDIP-58, Cervical Dystonia Impact Profile−58)*.

#### Sex Differences

There were no significant differences between men and women in age, duration of CD or years of formal education (Table 1). Men had an earlier median age at onset of CD than women (women: 45 years, men: 39 years; MWU = 3,369; *p* = 0.0062). TWSTRS-2 Total scores were marginally significantly worse for women than men [TWSTRS-2 Total (median, IQR) scores for women = 31 (20, 43); for men = 26 (21, 34); MWU = 3,639; *p* = 0.043)]; there were no significant sex differences in the TWSTRS-2 Pain, Disability and Severity scale scores (Table 1).

Women reported worse Health-Related Quality of Life (HrQoL) than men both by the Utility Index (*p* = 0.037) and by the CDIP−58 (*p* = 0.0088) (Table 1). All CDIP-58 subscale measures were larger (worse/more impact) in women than men but only significantly worse for the subscales “Sleep” (*p* < 0.038), “Upper limb symptoms” (*p* = 0.0002) and “Walking” (*p* = 0.012) (Table 2).

**Table 2:** Cervical Dystonia Impact Profile-58: Total scores and Subscale scores in 201 patients with cervical dystonia subdivided by sex and history of a mood disorder.

**Groups (*N*)**	**ALL (201)**	**WOMEN (136)**	**MEN (65)**	**History of mood disorder (79)**	**No h/o mood disorder (122)**
**CDIP-58 TOTAL**					
Mean (SD)	32.5 (21.9)	**35 (23)**	**26 (18)**	**38.5 (22.2)**	**28.6 (20.8)**
Median (IQR)	31 (15, 47)	**35 (17, 49)**	**23 (12, 38)**	**37 (21, 51)**	**24 (11.8, 44)**
		***(p = 0.0088)***		***(p = 0.002)***	
**HEAD AND NECK**					
Mean (SD)	**52.6 (27)**	55 (29)	48 (23)	**57.8 (25.0)**	**49.2 (27.7)**
Median (IQR)	**54 (33,75)**	57 (33, 78)	52 (33, 63)	**58 (42, 75)**	**50 (25,75)**
		*(p = 0.118)*		***(p = 0.042*****)**	
**PAIN**					
Mean (SD)	**44.9 (30.7)**	47 (32)	40 (27)	**51.2 (29.6)**	**40.9 (30.3)**
Median (IQR)	**45 (17.5,75)**	48 (20, 75)	35 (15,63)	**55 (25,75)**	**35(15,70)**
		*(p = 0.103)*		***(p = 0.0188)***	
**UPPER LIMB**					
Mean (SD)	31.4 (25.6)	**36 (26)**	**22 (22)**	**35.9 (25.9)**	**28.4 (25)**
Median (IQR)	28 (7, 51.5)	**33 (14, 56)**	**17 (0, 41)**	**33 (14, 56)**	**23.5 (5, 48)**
		***(p=0.0002)***		***(p = 0.038)***	
**WALKING**					
Mean (SD)	25.4 (26.3)	**29 (28)**	**18 (21)**	28.6 (27.9)	23.3 (25.2)
Median (IQR)	17 (0, 47)	**21 (1, 50)**	**14 (0, 30)**	17 (0, 50)	17 (0, 39)
		***(p = 0.0127)***		*(p = 0.234)*	
**SLEEP**					
Mean (SD)	26.7 (29.2)	**30 (30)**	**20 (26)**	**34 (30.3)**	**22 (27.6)**
Median (IQR)	19 (0, 50)	**19 (0, 50)**	**6 (0, 35)**	**31 (0, 56)**	**6 (0, 38)**
		***(p = 0.037)***		***(p = 0.0028)***	
**ANNOYANCE**					
Mean (SD)	28.5 (26.7)	31 (28)	23 (22)	**36.2 (28.4)**	**23.4 (24.4)**
Median (IQR)	19 (6, 50)	22 (6, 50)	16 (3, 43)	**34 (13, 53)**	**16 (3, 38)**
		*(p = 0.073)*		***(p = 0.0014)***	
**MOOD**					
Mean (SD)	23.9 (27.1)	26 (28)	19 (24)	**35.2 (30.7)**	**16.6 (21.7)**
Median (IQR)	11 (0, 39)	18 (4, 39)	7 (0, 38)	**25 (7, 57)**	**7 (0, 29)**
		*(p = 0.065)*		***(p< 0.0001)***	
**PSYCHOSOCIAL**					
Mean (SD)	34.7 (29.0)	37 (30)	30 (26)	**40.8 (30.4)**	**30.7 (27.4)**
Median (IQR)	28 (10, 54)	28 (13, 60)	23 (10, 45)	**33 (18, 68)**	**24 (10, 50)**
		*(p = 0.177)*		***(p = 0.0138)***	

*Cervical Dystonia Impact Profile−58 (CDIP-58): CDIP-58 Total and subscale scores [means, standard deviations (SD) medians and interquartile range (IQR)] in the study population of 201 patients. Comparison of CDIP-58 scores for 136 women and 65 men and also scores for the 79 patients with a history of a diagnosis of a mood disorder compared to 122 patients with no history of mood disorder. Women report significantly worse CDIP-58 Total, “Sleep,” “Upper Limb” and “Walking” subscale scores than men. A history of diagnosed mood disorder is associated with more disease impact in all measures except the “Walking” subscale. Group medians were compared by non-parametric, Mann Whitney testing; significant inter-group comparisons are illustrated in bold*.

#### History of Mood Disorder

**Figure 1:**
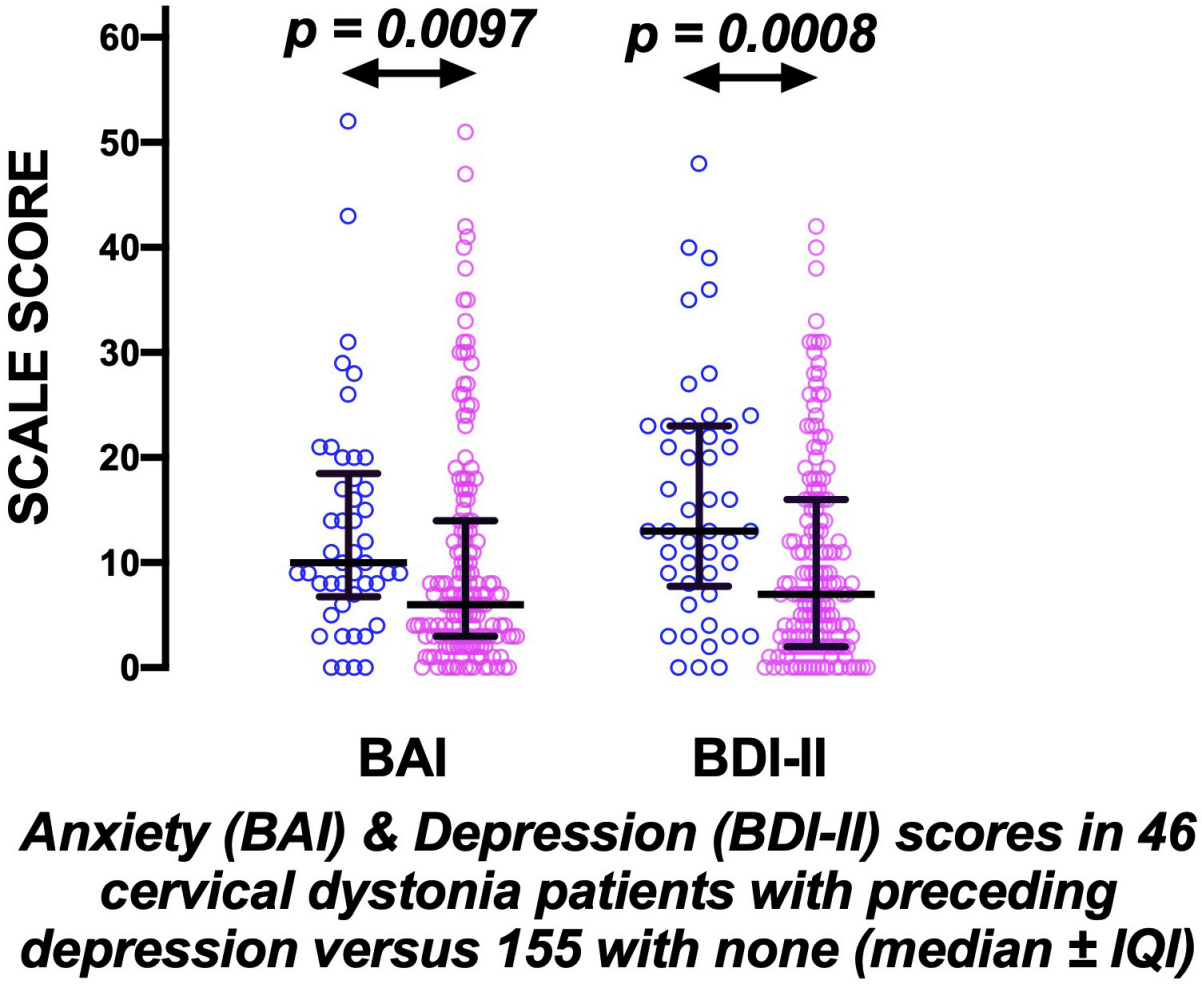
The prolonged effects of preceding mood disorder in cervical dystonia. Evidence of significant persisting anxiety and depression measured by the Beck Anxiety inventory (BAI) and Beck Depression Inventory (BDI-II) scores, ~18 years after disease onset, in 46 cervical dystonia patients (blue circles) with preceding anxiety/depression compared to 155 with none (pink circles) (bars indicate medians ± inter-quartile intervals).

46/201 (23%) patients had a history of a medical diagnosis of anxiety or depression prior to cervical dystonia onset; [35/136 women (26%) and 11/65 men (17%); NS]. These patients had persisting anxiety and/or depression; at review, their BAI and BDI-II scores remained markedly significantly worse than patients with no history of prior mood disorder, even after 15–20 years of dystonia (*p* = 0.0097 and *p* = 0.0008, respectively; Figure 1).

A history of a medically diagnosed mood disorder **at any stage** was reported by 79/201 (39%) (MD group); [58/136 women (42.6%) and 21/65 men (32.3%); NS]. In these 79 patients, compared to the 122 without such a history, the age of onset of cervical dystonia was 4 years earlier (*p* = 0.039) (Table 1). TWSTRS-2 Total scores were significantly worse in the 79 patients with a history of mood disorder (*p* = 0.03); this difference was driven by a markedly worse patient self-report of TWSTRS-2 Disability in these patients (*p* = 0.0067). As one might have anticipated, the 79 patients with a history of mood disorder reported significantly worse BAI and BDI-II scores (both *p* < 0.0001) (Table 1).

#### Current Mood Disorder Prevalence

Taking a very conservative measure of prevalent significant anxiety or depression, using the BAI (scores > 15) and the BDI-II (scores > 13), 37 /136 (27%) women had current anxiety, 49/136 (36%) had current depression, 53/136 (39%) had either anxiety or depression and 33/136 (24%) had both. For the 65 men 15 (23%) had current anxiety, 18 (28%) had current depression, 22 (34%) had either anxiety or depression and 11 (17%) had both.

### Determinants of Disease Impact by HrQoL Measures

#### Using CDIP-58 as the HrQoL Standard

##### Whole group analysis (Table 3 and Figure 2)

Within the whole group of 201 patients, using the CDIP-58 as the reference HrQoL measure, there were moderate correlations of the CDIP-58 with the BAI (*r*^2^ = 0.388), the BDI-II (*r*^2^ = 0.319), the TWSTRS−2 Total (*r*^2^= 0.386) and the patient reported TWSTRS-2 Pain (*r*^2^ =0.268) and Disability (*r*^2^= 0.289) subscales. The correlation of the CDIP-58 with the, physician-assessed, TWSTRS-2 Severity score was extremely low at *r*^2^ = 0.09. The BAI (*p* < 0.0001), the TWSTRS-2 Total (*p* < 0.0001), and subscales Pain (*p* < 0.0001) and Disability (*p* < 0.0001) survived multivariable analysis (Table 3).

**Table 3:** Determinants of Health-related Quality of Life in 201 patients with cervical dystonia.

**Dependent variable**	**TWSTRS-2 Pain**	**TWSTRS-2 Disability**	**TWSTRS-2 Severity**	**TWSTRS-2 Total**	**BAI**	**BDI-II**
	**R-squared relationship with the dependent variable**					
	**ALL 201 PTS**					
CDIP-58	0.268^****^	0.289^****^	0.09	0.386^****^	0.388^****^	0.319
Utility Index	0.110	0.200^***^	0.039	0.186^***^	0.367^****^	0.321^*^
**136 WOMEN**						
CDIP-58	0.291^****^	0.282^****^	0.116	0.421^****^	0.361^**^	0.302
Utility Index	0.070	0.158^**^	0.04	0.145^*^	0.319^**^	0.306*
**65 MEN**						
CDIP-58	0.17	0.30	0.056	0.307^**^	0.47^***^	0.36
Utility Index	0.238^**^	0.305^*^	0.020	0.34^**^	0.498^***^	0.349

Disease characteristic measures which are determinants of Health-related Quality of Life in 201 patients with cervical dystonia. Health-related Quality of Life (HrQoL) assessed by the Cervical Dystonia Impact Profile−58 (CDIP-58) and the EuroQoL Utility Index. Correlations (r-squared) between these HrQoL measures with patient reported levels of TWSTRS-2 Pain, TWSTRS-2 Disability, the physician-assessed TWSTRS-2 Severity; also, the Beck Anxiety Inventory (BAI) and the Beck Depression Inventory (BDI-II). Significant correlations, surviving multiple variable analysis, are in bold

(^*^ < 0.05; ^**^ < 0.01; ^***^ < 0.001; ^****^ < 0.0001) (TWSTRS, Toronto Western Spasmodic Torticollis Rating Scale).

**Figure 2:**
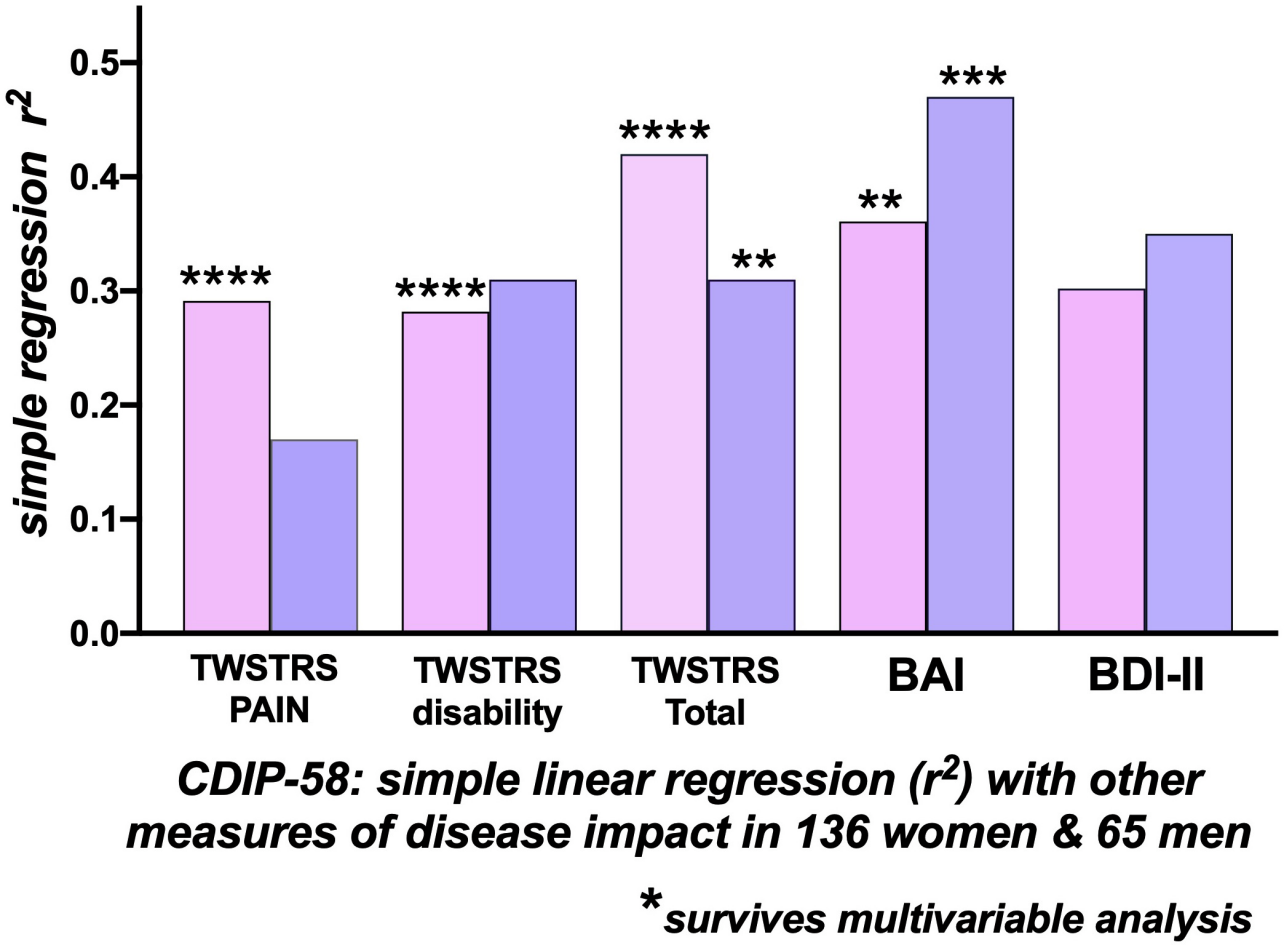
Correlations with the Cervical Dystonia Impact Profile-58 (CDIP-58) as an index of Quality of Life. Correlations of CDIP-58 with other measures of disease impact in 136 women (pink bars) and 65 men (blue bars) with cervical dystonia. The height of the bars indicates the r-squared relationship between the CDIP-58 Total Scale score and the clinical measure. In women there were significant correlations, surviving multi-variable analysis, between the CDIP-58 with TWSTRS-2 Total, Pain, Disability and Beck Anxiety Inventory (BAI). In men, only the TWSTRS Total and BAI correlations survived multi-variable analysis. [BAI, Beck Anxiety Inventory; BDI-II, Beck Depression Inventory; TWSTRS, Toronto Western Spasmodic Torticollis Rating Scale) (*p < 0.05; **p < 0.01; ***p < 0.001; ****p < 0.0001)].

##### Women

For the 136 women, the CDIP-58, as the dependent variable, correlated most strongly with the TWSTRS−2 Total (*r*^2^= 0.421), the BAI (*r*^2^= 0.361), BDI-II (*r*^2^= 0.302), the patient-reported TWSTRS−2 Pain (*r*^2^= 0.29) and TWSTRS−2 Disability scales (*r*^2^= 0.28); the correlation with the, physician assessed, TWSTRS−2 Severity score was low at *r*^2^= 0.11. The BAI (*p* = 0.0014), the TWSTRS−2 Total (*p* < 0.0001), Pain (*p* < 0.0001), and the TWSTRS−2 Disability subscales (*p* < 0.0001) survived multivariable analysis (Figure 2).

##### Men

For the 65 men, by single regression analysis, the most significant determinants of HrQoL, measured by the CDIP−58, were the BAI (*r*^2^= 0.47) and BDI-II (*r*^2^= 0.36); the TWSTRS-2 Total (*r*^2^= 0.31) and subscale Disability correlations were similar, the physician assessed, TWSTRS-2 Severity subscale correlation was extremely small. Only the BAI (*p* = 0.0006) and TWSTRS-2 Total (*p* = 0.0015) survived multivariable analysis (Table 3 and Figure 2).

#### Utility Index as the HrQoL Standard

##### Whole group analysis (Table 3)

For the 201 patients, the Utility Index, used as the reference HrQoL dependent variable, correlated most strongly with the BAI (*r*^2^ = 0.367), the BDI (*r*^2^ = 0.321), TWSTRS-2 Total (*r*^2^= 0.186) and the patient-reported TWSTRS-2 Disability scale (*r*^2^= 0.20). The correlation of Utility Index with the, physician-assessed, TWSTRS-2 Severity score was extremely low at *r*^2^ = 0.039. The BAI (*p* < 0.0001), BDI (*p* = 0.017) the TWSTRS-2 Total (*p* = 0.001) and TWSTRS−2 Disability scale (*p* = 0.001) survived multivariable analysis (Table 3).

##### Women

**Figure 3:**
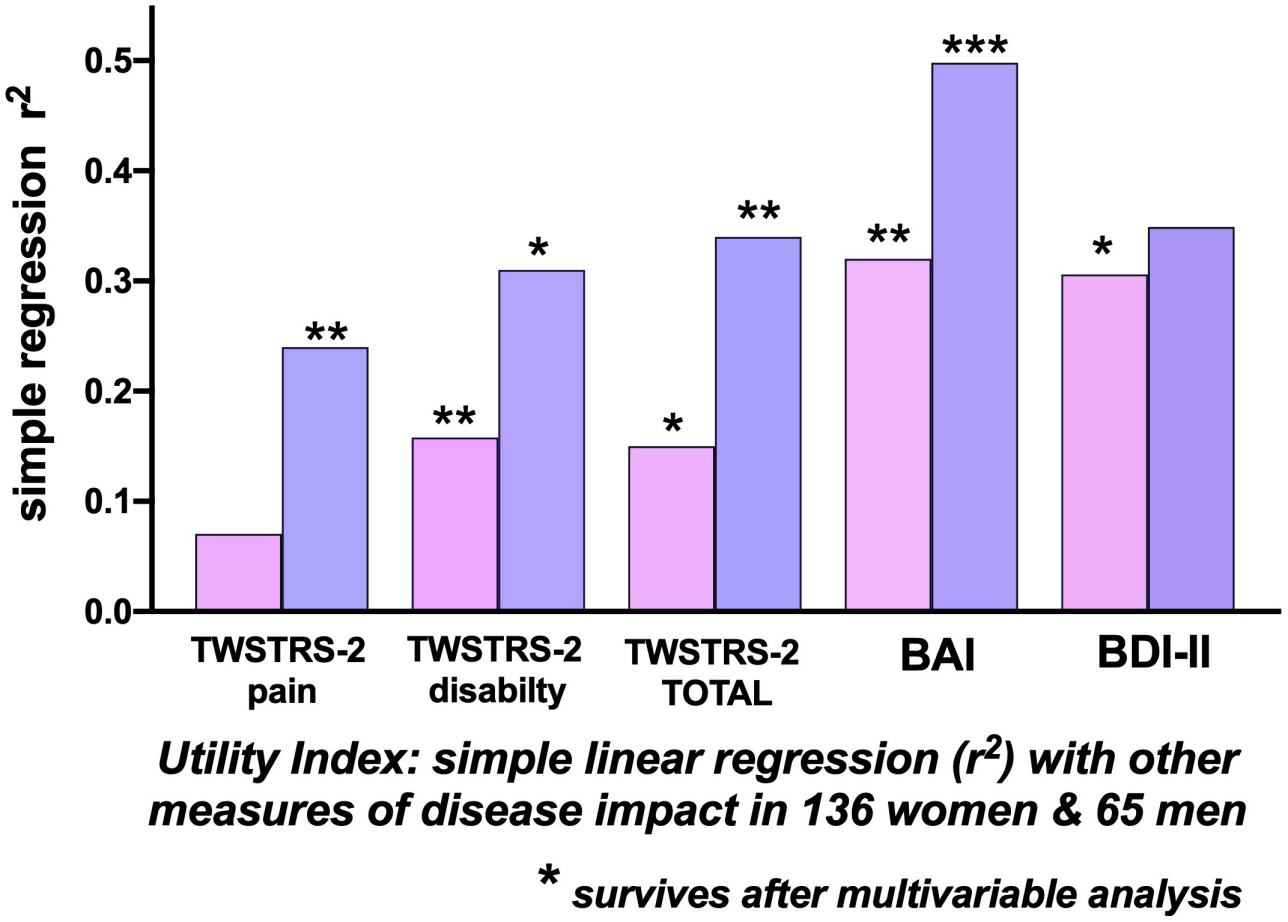
Correlations with the Utility Index as a measure of Quality of Life. Simple linear regression (*r*-squared) associations of the Utility Index with other measures of disease impact in 136 women (pink bars) and 65 men (blue bars) with cervical dystonia. The height of the bars indicates the univariate r-squared relationship between the Utility Index and the clinical measure. In 136 women, the TWSTRS-2 Total, TWSTRS-2 Disability subscale, Beck Anxiety Inventory, and Beck Depression Inventory (BDI-II) correlations survived multi-variable analysis. In 65 men there were significant separate correlations, surviving multi-variable analysis, between the Utility Index and the TWSTRS-2 Total, TWSTRS-2 Pain, and Disability subscales and with the Beck Anxiety Inventory (BAI). [TWSTRS, Toronto Western Spasmodic Torticollis Rating Scale; BAI, Beck Anxiety Inventory; BDI-II, Beck Depression Inventory; (*p < 0.05; **p < 0.01; ***p < 0.001.

For the 136 women, the Utility Index as the dependent variable, correlated most highly with the BAI (*r*^2^ = 0.319) and the BDI-II (*r*^2^ = 0.31); the correlation with the TWSTRS−2 Total was much weaker at *r*^2^ = 0.145; most of this correlation related to the TWSTRS−2–Disability subscale (*r*^2^ = 0.158); the physician assessed TWSTRS−2 Severity correlation was extremely weak at *r*^2^ = 0.04 (Table 3). The BAI (*p* = 0.007), the BDI-II (*p* = 0.02) and the TWSTRS−2 Disability subscale (*p* = 0.007) survived multivariable analysis (Figure 3); The TWSTRS-2 Total also survived (*p* = 0.047) when assessed separately from the subscales (and clearly was driven by Disability subscale).

##### Men

For the 65 men, the highest correlations of the Utility Index were with the BAI (*r*^2^= 0.498), the BDI-II (*r*^2^= 0.349) and the TWSTRS−2 Total (*r*^2^= 0.34). Correlations within the TWSTRS−2 subscales were: Disability (*r*^2^= 0.31) and Pain (*r*^2^= 0.24); the, physician-assessed, Severity correlation was *r*^2^= 0.03 (Table 3). The BAI (*p* = 0.0003), TWSTRS−2 Total (*p* = 0.004), Pain (*p* = 0.003) and Disability subscales (*p* = 0.03) survived multivariable analysis (Figure 3).

## Discussion

In this study, we found that HrQoL in cervical dystonia, assessed by both a generic measure (EuroQoL Utility Index) and a disease specific instrument, the CDIP-58, is overwhelmingly determined by a combination of factors: anxiety, depression and the patient's report of pain and disability (TWSTRS-2 Pain and TWSTRS-2 Disability). Non-motor symptoms, in particular mood disorder, were the predominant determinants of the patient's HrQoL, as has been indicated by others (discussed below). Factors which did not show correlation with HrQoL in our study included, duration of cervical dystonia, years of formal education, and current age.

The size of the variances of measures of mood, particularly anxiety, indicates that at least 40–50% of HrQoL impairment may be attributable to a psychological disorder intrinsic to cervical dystonia which is not currently addressed in the clinic. One of the problems of a busy botulinum toxin clinic, with repeated 3-monthly injections, is that the patient-doctor interaction is directed purely at the mechanical injection process and not with the unspoken, and not enquired about, considerable psychological morbidity.

One noteworthy aspect of this study is that disease severity, assessed by the neurologist using the TWSTRS-2 Severity scale, on univariate analysis, had a negligible contribution to HrQoL in comparison to the patient-reported measures TWSTRS-2 Pain and TWSTRS-2 Disability. Both the latter were the most significant contributors to the TWSTRS−2 Total score and most relevant to HrQoL, either measured by the Utility Index or the CDIP-58. The TWSTRS Severity scale has been noted to be insensitive to change; in an assessment of minimally important clinical change in the TWSTRS score (CD PROBE) it was reported that the Severity subscale was least useful of all the three subscales (29); a comprehensive review of rating scales indicates the difficulties in measuring cervical dystonia severity, not all which can be overcome by ensuring good inter-rater reliability (30). A recent multidimensional assessment of HrQoL and psychiatric morbidity in cervical dystonia also found no effect of TWSTRS-2 Severity assessment (12). It could be argued we should discontinue using physician assessment of disease severity by the TWSTRS-2 methodology, especially in CD patients already established on long-term botulinum toxin therapy; we should pay more attention to patient report from measures such as the TWSTRS-2 Disability, Pain and the CDIP-58. It is possible that the future technological development of wearable devices to assess the severity of motor symptoms over the period of several days prior to the clinic visit, might be a useful objective measure of the severity of motor symptoms; at present it would appear that the summary expert neurologist's assessment of disease severity at the clinic visit is less than useful.

### Our Findings in the Context of Previous Research

There have been, over the last 10 years, a number of comprehensive reviews of non-motor symptoms (including psychiatric symptoms) in adult onset focal dystonia (3, 7, 31). There have also been a number of studies using relatively few participants (14) and with a mixture of various phenotypes of focal dystonia, which makes comparison to the present study difficult.

The Epidemiological Study of Dystonia in Europe Collaborative Group was one of the first to assess HrQoL, using the SF-36, in a number of participants with cervical dystonia; in a postal survey of 286 European patients with cervical dystonia, men reported better HrQoL scores than women, which were significantly different in relation to pain and “physical function” (32). When compared to other neurological conditions including stroke, multiple sclerosis and Parkinson's disease, patients with cervical dystonia scored worst in relation to HrQoL. Using the same database, and with measures of mood, a further paper noted that, following multivariable analysis, the strongest predictors of HrQoL in this population were depression and anxiety, although self-reported severity remained a significant variable; it was noted that longer disease duration was a significant ameliorating factor in reported HrQoL (4).

A Polish study of 101 patients with cervical dystonia on treatment with botulinum toxin noted that depression (found in 47% of patients) was the main predictor of poorer HrQoL; women reported poorer quality of life (33). Surprisingly, not in keeping with our findings, they noted that longer treatment duration was associated with a beneficial effect; given that this study was carried out in the first few years after the introduction of botulinum toxin, this beneficial effect might be attributable to a comparison with the physical state prior to its introduction. A Norwegian study in 2007 assessed 70 patients with cervical dystonia using TWSTRS, HADS-D and a quality of life measure, the SF-36; they found that mean SF 36 scores in the patients were reduced by one standard deviation compared to the general population; the main factors associated with reduced scores in the physical and mental domains of the SF 36 was the TWSTRS Total and the HADS-D scores, respectively (13). Surprisingly, compared to other reports they found that patients with a good response to botulinum toxin had both lower TWSTRS scores and less depression. A German study in 2009, in 86 patients (predominantly cervical dystonia) noted an increased risk of various psychiatric disorders including depression with an odds ratio of 3.0 and 21.6 for social phobia (10). A postal survey study in 2009 from Sweden of 279 cervical dystonia patients using the CDIP-58 reported similar but slightly higher, scores in all of the subscales than we found; there was no concurrent measure of mood disorder or generic quality of life to compare with our patient sample (16).

There was a very influential case-control Italian study of 89 patients with various forms of focal dystonia (34 had cervical dystonia) from Fabbrini et al. (8). They used 62 healthy control participants; 64% of the cervical dystonia patients had a psychiatric disorder compared to 25% of healthy controls; this was due to an increased rate of major depression; the prevalence of anxiety was not increased; in 15/22 (68%) cervical dystonia patients with a psychiatric disorder the psychiatric disorder had begun prior to the movement disorder (8). A follow-up to that paper noted that, when reassessed 5 years later, the patients had not improved from the point of view of the psychiatric symptoms, although the severity of their dystonia was milder at the second assessment (5). The authors concluded that the psychiatric symptoms were independent of the motor symptomatology.

An important Dutch case-control study intensively assessed psychiatric morbidity in 50 patients with cervical dystonia, the cervical dystonia population had a 64% prevalence of psychiatric disorders compared to 28% in a control population; depression prevalence was 32% vs. 14%; anxiety 42% vs. 8%; other psychiatric disorders were not increased in frequency (12). They also found, similar to our results, that, following multivariable analysis, HrQoL was strongly predicted by high scores on the BDI-II, BAI, TWSTRS-2 Disability and TWSTRS-2 Pain. TWSTRS-2 Disability was significantly associated with the severity of depression measured by the BDI-II (*r*^2^ = 0.28); TWSTRS-2 Pain was significantly associated with TWSTRS-2 Disability and with anxiety measured by the BAI (*r*^2^ = 0.32). Smit et al. also noted, similar to our findings, that dystonia motor severity (by TWSTRS-2 Severity) had no influence on disability, pain or HrQoL (12).

A recent, very comprehensive, report from the Dystonia Coalition noted that 32.8% of their 255 cervical dystonia patients (from multiple centers) had depression by the BDI-II (>13) and 43.8% had anxiety by the HADS-A (>7); they noted that their data confirmed the large and growing body of evidence of the coexistence of depression and anxiety with isolated focal dystonia (6).

In our study, we found a high prevalence of anxiety and/or depression based on a medically diagnosed history (almost 40%), and on using assessment tools (about 40%); this supports the accumulating evidence of the high prevalence of anxiety and depression in dystonia patients (12–14, 17, 18). While depression (by BDI-II and a known history) is more prevalent than anxiety (by BAI and a known history) in our study, anxiety (by BAI) showed more impact on quality of life than depression (by BDI-II); this has not been described before.

The strengths of our study include the homogeneity of the dystonia phenotype and size of our single-center, cohort, together with the 96% response rate in participation. One limitation of our study is the inevitable retrospective reporting by our patients of the presence, and time of onset, of mood disorder relative to the onset of their cervical dystonia, although this would have been recorded in their medical notes at their initial consultation.

## Conclusions

Patient-reported measures, particularly the CDIP-58, are more sensitive and reflective of health-related quality of life than the physician-administered standard recommended measure of cervical dystonia severity in patients receiving botulinum toxin therapy. The ability to control motor symptoms of cervical dystonia using botulinum toxin therapy has major benefits. However, recognizing and addressing anxiety and depression in these patients is necessary to improve their quality of life. There is an increasing consensus that psychiatric symptoms, anxiety and depression, are a primary symptom of adult onset dystonia (not secondary to the movement disorder). It is considered that these psychiatric symptoms indicate disordered processing within limbic–striatal systems and are due to the same underlying processes, which cause the movement disorder.

The logical imperative is that movement disorders neurologists must address these psychiatric symptoms and resulting disability; although a recent short duration randomized control study of escitalopram was negative (34), longer duration, randomized-controlled trials of SSRI therapy in cervical dystonia are warranted.

## Data Availability Statement

The raw data supporting the conclusions of this article will be made available by the authors, without undue reservation.

## Ethics Statement

This study was approved by St Vincent's Healthcare Group, Ethics and Medical Research Committee (no reference number assigned). Written informed consent was obtained from all participants of the study. We confirm that we have read the Journal's position on issues involved in ethical publication and affirm that this work is consistent with those guidelines. The patients/participants provided their written informed consent to participate in this study.

## Author Contributions

IN: organization and execution of research project. SO'R: execution of research project. MH: conception and organization of research project. IN and MH: design and execution of statistical analysis, writing of the first draft, and review and critique of the manuscript. CW: review and critique of statistical analysis. SO'R and CW: review and critique of the manuscript. All authors: contributed to the article and approved the submitted version.

## Conflict of Interest

The authors declare that the research was conducted in the absence of any commercial or financial relationships that could be construed as a potential conflict of interest.
